# Non-epidemic HCV genotypes in low- and middle-income countries and the risk of resistance to current direct-acting antiviral regimens

**DOI:** 10.1016/j.jhep.2021.04.045

**Published:** 2021-08

**Authors:** Rajiv Shah, Lucrece Ahovegbe, Marc Niebel, James Shepherd, Emma C. Thomson

**Affiliations:** 1 MRC-University of Glasgow Centre for Virus Research, Glasgow, UK; 2 Mbarara University of Science and Technology, Mbarara, Uganda; 3 London School of Hygiene and Tropical Medicine, London, UK

**Keywords:** Hepatitis C Virus, Genotypes, Subtypes, Directly Acting Antivirals, SVR, Resistance, Elimination, Sequencing

## Abstract

The hepatitis C virus (HCV) is an extremely diverse virus, subtypes of which are distributed variably around the world. Viral genotypes may be divided into epidemic subtypes; those that have become prevalent globally, and endemic subtypes that have a more limited distribution, mainly in Africa and Asia. The high variability of endemic strains reflects evolutionary origins in the locations where they are found. This increased genetic diversity raises the possibility of resistance to pan-genotypic direct-acting antiviral regimens. While many endemic subtypes respond well to direct-acting antiviral therapies, others, for example genotypes 1l, 3b and 4r, do not respond as well as predicted. Many genotypes that are rare in high-income countries but common in other parts of the world have not yet been fully assessed in clinical trials. Further sequencing and clinical studies in sub-Saharan Africa and Asia are indicated to monitor response to treatment and to facilitate the World Health Organization’s 2030 elimination strategy.

## Introduction

**Fig. 1:**
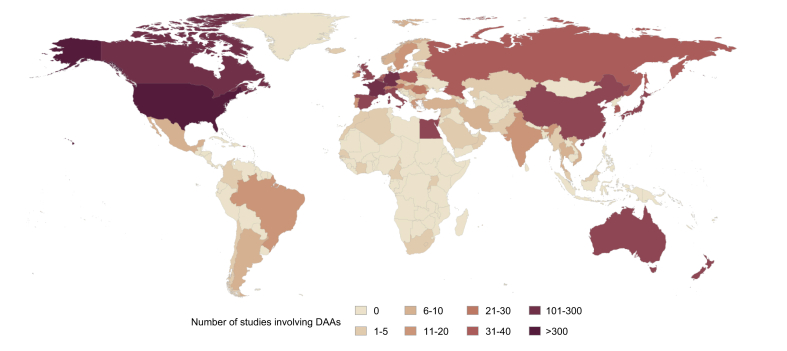
Map showing locations of DAA-related studies involving HCV-infected individuals. Details of registered clinical trials were downloaded from clinicaltrials.org. DAA, direct-acting antiviral.

The revolution in therapeutic options for the treatment of the hepatitis C virus (HCV) is one of the most important medical advances in a generation and has led the World Health Organisation (WHO) to propose a plan for elimination by 2030. Sustained virological response (SVR) rates of more than 95% have become the norm in high-income countries (HICs), using treatments that have very few side effects and are cheap to manufacture, albeit subject to country-specific pricing differences.^1^^,^^2^ However, a key weakness of the clinical research underpinning this plan is that almost all of it was carried out in HICs, a weakness repeatedly acknowledged by the international WHO HCV treatment guidelines committee, that focuses on the needs of low- and middle-income countries (LMICs) (Fig. 1).^3^ Early indications that SVR rates might not always be as high as expected in some populations emerged from studies on direct-acting antiviral (DAA) treatment in Europe and North America that found diverse genotypes in patients originating from sub-Saharan Africa.[4], [5], [6] Subsequently, emerging studies investigating response rates to DAAs in Africa and Asia have confirmed these initial reports on a larger scale.^7^

HCV is one of the most genetically diverse human viruses.^8^ This genetic diversity, which varies by geographical region, has not yet been fully characterised, due to under-sampling in many parts of the world, meaning that the likelihood of drug resistance in many viral subtypes has not been assessed. At the time of writing, 8 genotypes and 90 subtypes of HCV have been described with an average pairwise distance of more than 30% between genotypes and an intra-genotype average pairwise distance of 15% between subtypes.^8^ These genotypes may be divided into genetically conserved epidemic lineages that have been exported large scale around the world and more localised highly diverse endemic lineages. Several endemic lineages have recently been found to be associated with resistance to some “pan-genotypic” DAA regimens (in particular those containing first-generation NS5A inhibitors) and may represent a threat to the global elimination plans proposed by the WHO.

**Key point**

HCV is an extremely diverse RNA virus with 8 known genotypes and 90 known subtypes.

Response to treatment *in vitro* and in clinical trials in epidemic genotypes prevalent in Europe, the United States, North Africa, parts of Asia and the Indian sub-continent (genotypes 1a,1b, 2a, 3a, 4a, 4d and 6a) has been studied in detail. DAAs with activity against such genotypes have been termed “pan-genotypic” and have enabled the WHO to develop a series of guidelines aimed at elimination of HCV by 2030.^3^ These guidelines aim to simplify treatment regimens so that genotyping (which is expensive and can provide a barrier to treatment, particularly in LMICs) is not required at the individual level.

In this review article, we make the case that there is a need for further population-based sequencing and real-world clinical studies to assist policymakers in individual countries to select more nuanced treatment strategies appropriate to the local setting, given emerging evidence that some endemic genotypes present in sub-Saharan Africa and Asia may respond less well than originally anticipated to DAAs, particularly first-generation NS5A inhibitors such as daclatasvir and ledipasvir.

## Methods in brief

A comprehensive review was carried out based on searching the literature for all endemic genotypes (excluding the epidemic genotypes 1a,1b, 2a, 3a, 4a, 4d and 6a). The primary source of genetic sequence data and published references was HCV GLUE,^9^ which is a comprehensive dataset of HCV genetic sequences with associated metadata from the NCBI. The sequences and metadata are curated and organised into genotypes and subtypes with linked relevant PubMed references. The sequences are linked to a database of polymorphisms known to be associated with DAA treatment failure or reduced DAA efficacy *in vitro*, developed and maintained by an expert resistance group led by Public Health England.

A supplemental literature search was conducted on PubMed to search for every individual endemic sub-genotype, *e.g*. “4r” and “treatment” and any additional studies describing HCV genetic diversity and related geographic distribution as well as reported treatment outcomes using the following search terms: “genotype”, “rare”, “unusual” or “uncharacterised” and “HCV” or “hepatitis C virus” and “treatment” or “management” or “direct-acting antiviral” or “DAA” or “interferon-free”.

To show the number and location of completed and ongoing DAA treatment studies, data were obtained from www.clinicaltrials.gov. Included studies were not limited to randomised controlled trials, provided DAAs were administered to HCV-infected individuals in the country or countries where the study was conducted. Studies involving only healthy volunteers and studies involving use of the first-generation protease inhibitors telaprevir and boceprevir with pegylated-interferon and ribavirin were excluded. A further 2 studies were excluded as no information on the country where the study was conducted could be found.

Data manipulation was carried out in R (version 3.5.3).

## Geographical distribution of genotypes

**Fig. 2:**
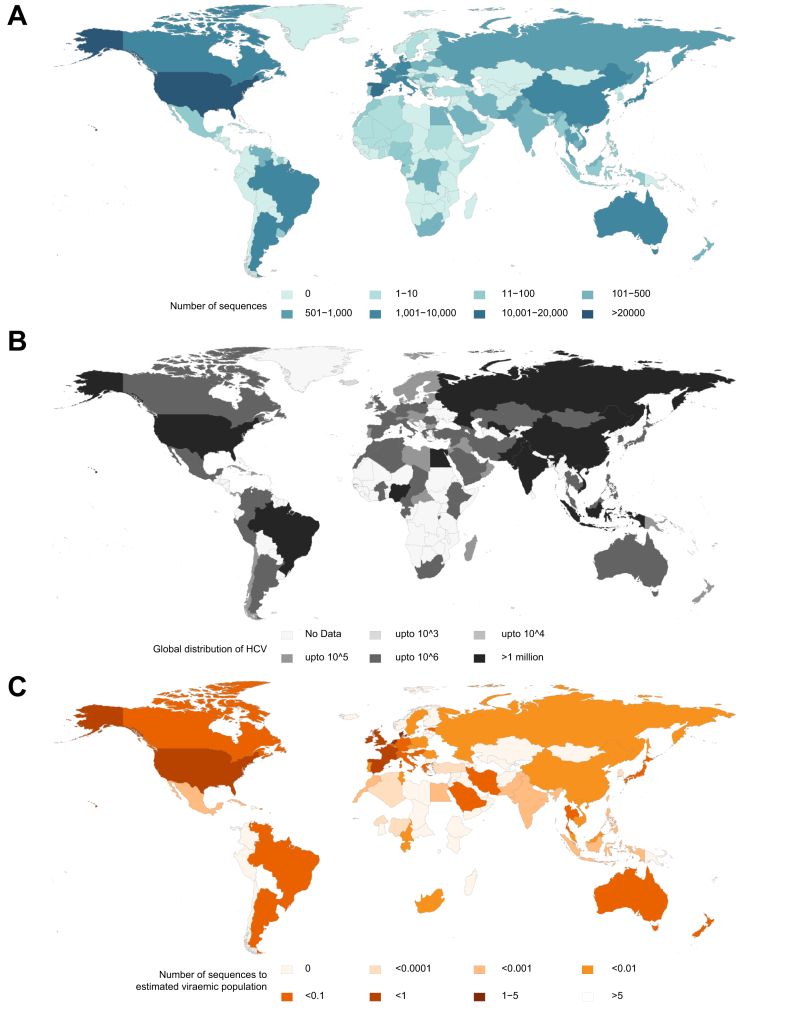
Global maps illustrating availability of sequence data, distribution of viraemic population and ratio of sequence data to infected population. (A) HCV genetic sequences greater than 500 nucleotides in length uploaded to GenBank and curated using HCV GLUE by country (accessed on 4^th^ August 2020). (B) Global distribution of estimated HCV viraemic population.^50^ (C) Ratio of the number of published HCV genetic sequences to the estimated viraemic population per country.

The distribution of HCV genotypes around the world is highly heterogenous, with the highest levels of diversity in Asia and sub-Saharan Africa; paradoxically, these regions are the least sampled (Fig. 2). The high genetic diversity of HCV in these locations most likely reflects the evolutionary origins of HCV subtypes. In contrast, the more heavily sampled but far less diverse epidemic lineages of HCV (including 1a, 1b, 2a, 2b, 2c, 3a, 4a, 4d and 6a), most likely represent a diaspora of exported founder strains that dispersed rapidly due to medical and recreational use of injections, blood transfusions, and operative medical procedures, peaking during the 20^th^ century. The coincidence of industrialisation alongside changes in medical care and an increase in wealth in some countries has left a legacy of highly sampled relatively conserved epidemic strains in HICs and a highly diverse but largely invisible pool of HCV in LMICs. HCV lineages associated with increased intrinsic resistance to the NS5A inhibitors, with different levels of resistance according to the drug and drug generation, are harboured within this reservoir of endemic strains and could present a barrier to global elimination if transmission is not prevented in affected areas.

**Key point**

10 epidemic lineages (1a, 1b, 2a, 2b, 2c, 3a, 4a, 4d, 5a, 6a) are well described yet there are many endemic lineages that are poorly characterised clinically, but highly prevalent, particularly in low middle-income countries.

**Fig. 3:**
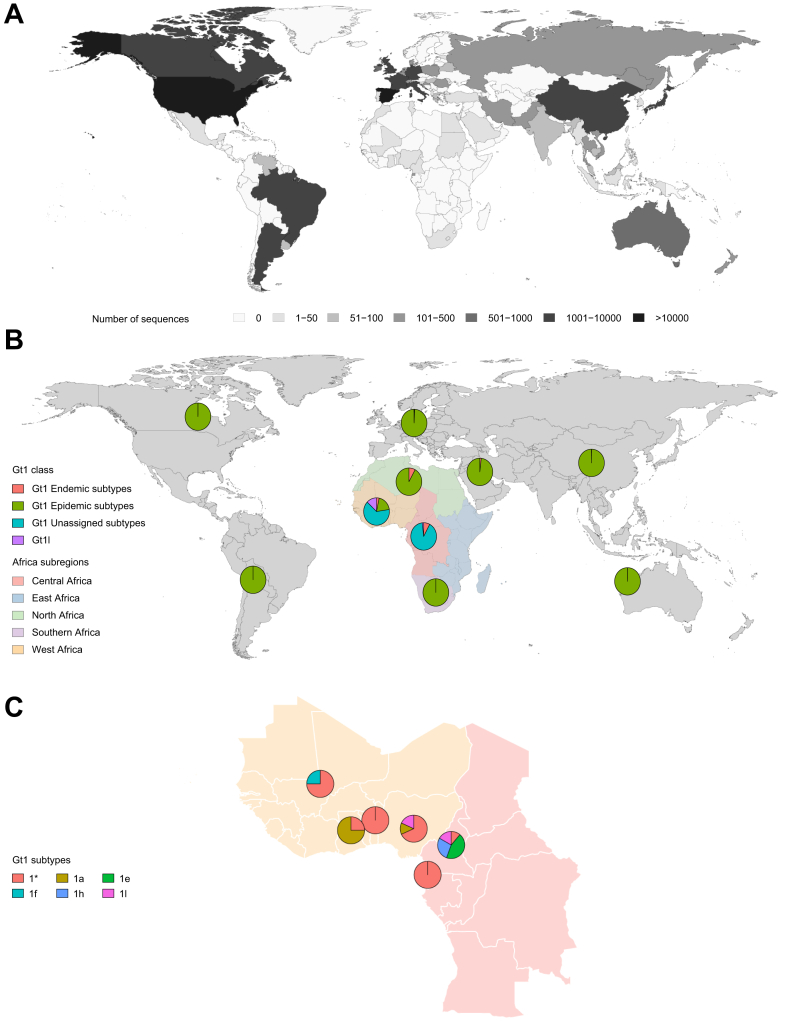
Genotype 1 genotype and sub-genotype distribution. (A) Global distribution of genotype 1 HCV. (B) Genotype 1 subtype distribution with a focus on the subregions of Africa where genotype 1 is most diverse. Epidemic genotype 1 subtypes include 1a and 1b. Endemic genotype 1 subtypes include 1c, 1d, 1e, 1f, 1g, 1h, 1j, 1k, 1l and 1m. (C) Genotype 1 subtype diversity within West Africa (Benin, Ghana, Mali and Nigeria) and Central Africa (Cameroon and Equatorial Guinea). 1∗ refers to unassigned Genotype 1 subtypes.

Genotype 1a and 1b are the most widespread genotype 1 lineages, distributed around the globe by the founder effect (Fig. 3) through the use of injections in people who inject drugs (PWID) and within healthcare settings,^10^ and to a far lesser extent through sexual^11^ and vertical transmission.^12^ Genotype 1 is likely to have originated in West Africa where extremely high levels of sub-genotypic diversity are evident.^13^

**Fig. 4:**
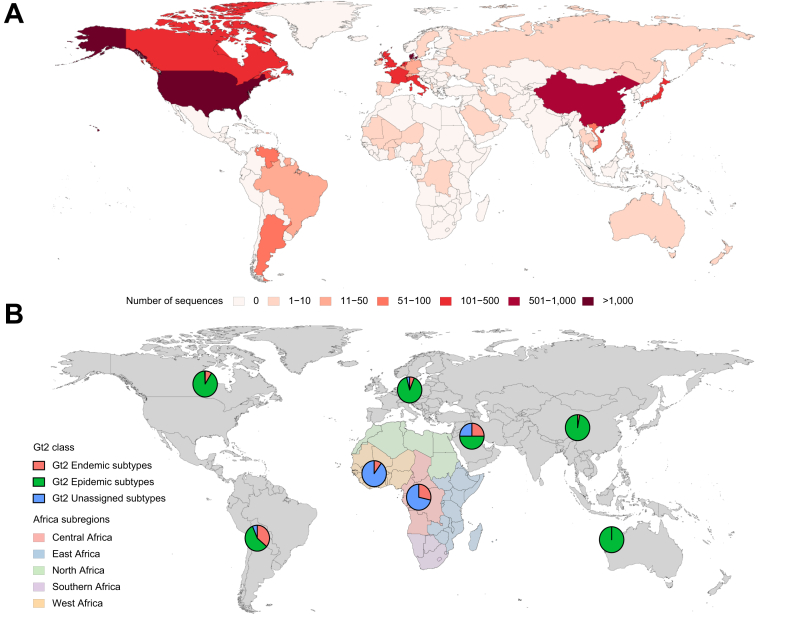
Genotype 2 genotype and sub-genotype distribution. (A) Global distribution of Genotype 2 HCV. (B) Genotype 2 subtype distribution with a focus on the subregions of Africa where it is most diverse. Epidemic Genotype 2 subtypes include 2a, 2b and 2c. Endemic Genotype 2 subtypes include 2d, 2e, 2f, 2i, 2j, 2k, 2l, 2m, 2n, 2o, 2q, 2r, 2s, 2t and 2u.

Genotype 2 is also likely to have originated in West Africa^14^ where it is most prevalent and most diverse (Fig. 4). It dispersed widely within West African countries most likely as a result of iatrogenic blood-borne transmission, for example during military medical public health interventions that aimed to treat the whole population of French Cameroon between 1921 and 1957 for African trypanosomiasis, yaws, syphilis and leprosy using injected treatments. Genotype 2 has also spread to other parts of the world. It was exported to the Caribbean by sea as a result of the transatlantic slave trade; sequences sampled in Martinique closely resemble those from the Benin–Ghana area (people in Martinique are known to share ancestry with populations in present-day Ghana, Togo, Benin and Nigeria). Genotype 2 also migrated from South America to Asia, likely at least in part via the slave trade between Indonesia and Suriname. Reflecting this history, genotype 2 strains are also found in the Americas, for example in the USA (2a,2b), Argentina (2a,2c), Venezuela (2b,2c, 2j, 2s), Suriname (2e,2f,2j,2o), Brazil (2a,2b,2c) and Hispaniola (2r) and in Asia – Indonesia (2e), China (2a), Japan (2a,2b), Thailand and Vietnam (2a, 2m).^15^

**Fig. 5:**
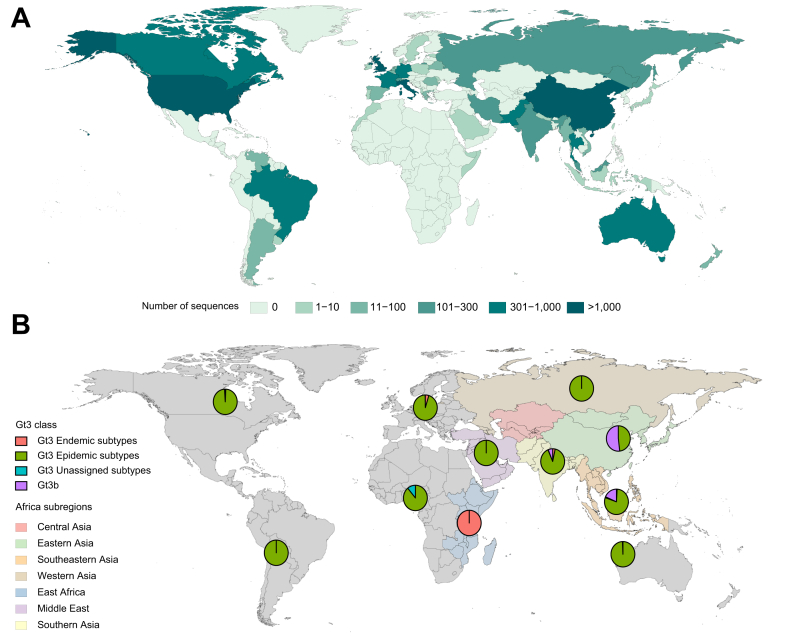
Genotype 3 genotype and sub-genotype distribution. (A) Global distribution of Genotype 3 HCV. (B) Genotype 3 subtype distribution with a focus on the subregions of Asia and East Africa where it is most diverse. The epidemic Genotype 3 subtype is 3a. Endemic Genotype 3 subtypes include 3d, 3e, 3f, 3g, 3h, 3i and 3k.

Genotype 3 is highly prevalent in several regions of the world (Fig. 5), with most heterogeneity in the Indian sub-continent and Southeast Asia.^16^ The majority of trials have focused on the epidemic genotype 3a subtype that is prevalent in the Indian sub-continent and has most successfully entered populations in HICs. However, genotype 3b^17^ is also distributed widely in Asia, including India, China, Malaysia and Thailand. The less well characterised genotypes 3c (provisional), 3d and 3e have been found in Nepal, 3f, 3g and 3i in India and 3k in Indonesia.^18^ Genotype 3 strains are uncommonly detected in South and Central America and in sub-Saharan Africa, but exist in East Africa where genotype 3h has been found in Somalia and in individuals originating from this area.^19^

**Fig. 6:**
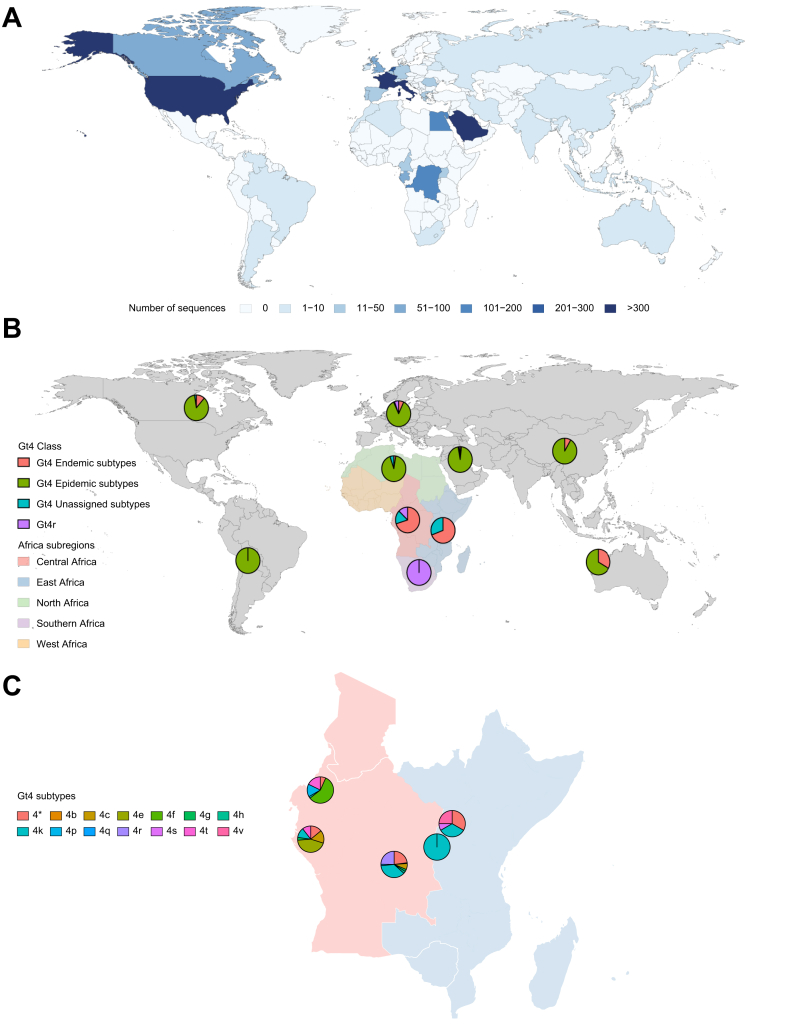
Genotype 4 genotype and sub-genotype distribution. (A) Global distribution of Genotype 4 HCV. (B) Genotype 4 subtype distribution with a focus on the regions of Africa where it is most diverse. Epidemic Genotype 4 subtypes include 4a and 4d. Endemic Genotype 4 subtypes include 4b, 4c, 4e, 4f, 4g, 4h, 4i, 4k, 4l, 4m, 4n, 4o, 4p, 4q, 4s, 4t, 4v and 4w. (C) Genotype 4 subtype diversity within Central (Cameroon, Democratic Republic of Congo and Gabon) and East Africa (Rwanda and Uganda). 4∗ refers to unassigned Genotype 4 subtypes.

Genotype 4 HCV (Fig. 6) is likely to have originated in Central and East Africa where multiple diverse endemic strains predominate.^20^ Several lineages have spread into North Africa and further afield, of which genotypes 4a and 4d are most dispersed. Genotype 4a is the most prevalent genotype 4 strain around the world and transmission was amplified in North Africa, particularly Egypt as a result of unsafe injection practices in healthcare settings.^21^ Clinical trials have focused on this subtype, and to a lesser extent genotype 4d, a strain introduced to Saudi Arabia during the early 20^th^ century,^22^ probably from countries in the Horn of Africa including Ethiopia.^23^ It has more recently emerged in PWID in Southern Europe and found a sexually-transmitted niche in men-who-have-sex-with-men (MSM) in Northern Europe with cross-over from PWID to MSM likely occurring in urban centres such as Amsterdam, London, Berlin and Paris.^24^ While the globally dispersed genotype 4a and 4d lineages are well described, others prevalent in sub-Saharan Africa have only recently been investigated in clinical studies.

**Key point**

Asia and sub-Saharan Arica host the most diverse HCV lineages but are the least well sampled for genomic data.

**Fig. 7:**
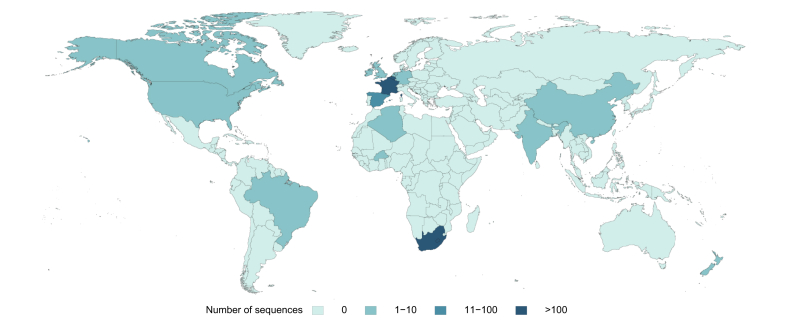
Genotype 5 genotype and sub-genotype distribution. Global distribution of Genotype 5 HCV. The shading in Asia represents a full length and one partial HCV sequence from China^51^ as well as one full length HCV sequence from India (unpublished), reflecting how rare Genotype 5 HCV is in Asia.

Genotype 5 is the most common genotype in South Africa^25^ and has been exported to Europe, Asia and North America in rare cases (Fig. 7). There is currently 1 official subtype (5a), however a second highly divergent lineage has been identified in Burkina Faso,^26^ suggesting that genotype 5 may be more widespread on the African continent than previously suspected (official sub-genotypes require 3 separate sequences from at least 3 infected individuals to be officially confirmed).^8^

**Fig. 8:**
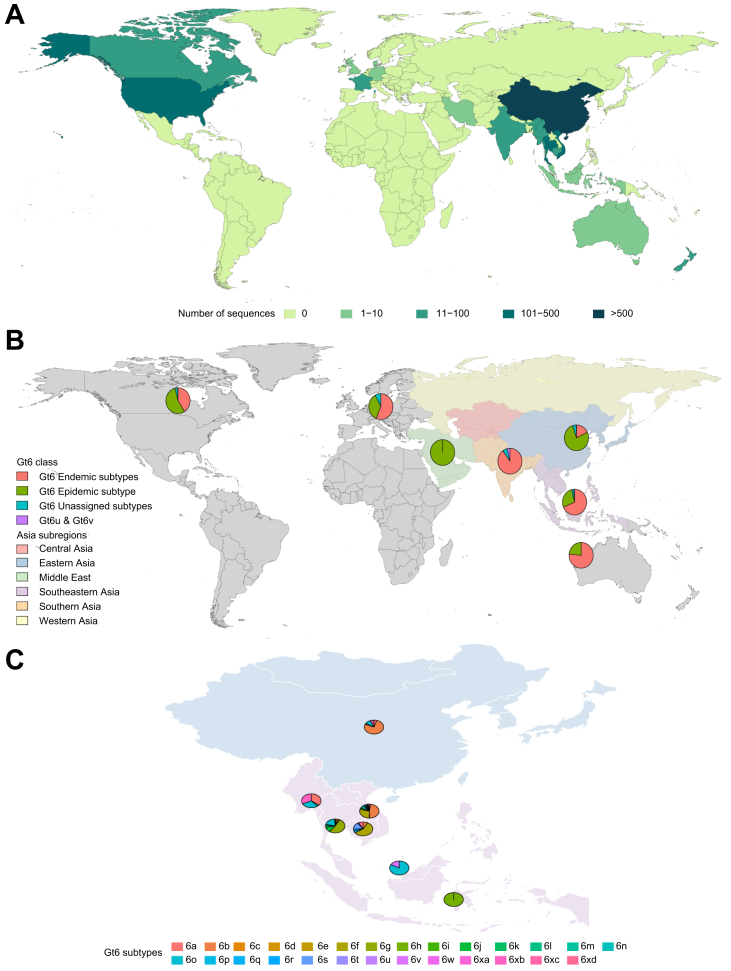
Genotype 6 genotype and sub-genotype distribution. (A) Global distribution of Genotype 6 HCV. (B) Genotype 6 subtype distribution with a focus on the regions of Asia where they are most diverse. The epidemic Genotype 6 subtype is 6a. Endemic Genotype 6 subtypes include 6b, 6c, 6d, 6e, 6f, 6g, 6h, 6i, 6j, 6k, 6l, 6m, 6n, 6o, 6p, 6q, 6r, 6s, 6t, 6w, 6xa, 6xb, 6xc, 6xd, 6xe. (C) Genotype 6 subtype diversity within Eastern (China) and South-eastern Asia (Cambodia, Thailand, Vietnam, Myanmar, Malaysia and Indonesia). 6∗ refers to unassigned Genotype 6 subtypes.

Genotype 6 is the most diverse HCV genotype and is most prevalent in Southeast Asia (Fig. 8) where further geographic heterogeneity of subtypes is evident.^27^ While the epidemic strain 6a has been exported widely around the world, other subtypes are far more spatially structured within distinct geographical locations. Genotype 6u is found in Laos^28^ and Vietnam^29^ while 6v has been detected in China and Thailand.^30^^,^^31^

Genotypes 7 and 8 have only recently been identified in individuals originally from the DRC, Uganda^32^ and India.^33^ The distribution of these subtypes requires further investigation.

## Susceptibility to DAAs in non-epidemic HCV subtypes

Reduced efficacy of DAAs is most often related to polymorphisms within the NS5A gene in epidemic subtypes of HCV and this also appears to be the case with endemic strains.^2^^,^^3^^,^^34^ A list of polymorphisms predicted to confer resistance is included in Table S1. This has been well-documented with the first-generation NS5A inhibitors ledipasvir and daclatasvir but may also affect SVR rates with second-generation inhibitors. Amino acid polymorphisms associated with resistance within NS3 have been reported in endemic strains but have not been tested extensively in clinical trials. Polymorphisms in NS5B are not common – the well described but uncommon and unfit S282T polymorphism is associated with reduced efficacy of sofosbuvir in epidemic lineages. This has occasionally been detected in subtypes 4r and 6l but the *in vivo* impact is unknown.^35^^,^^36^

Resistance-associated substitutions (RASs) in epidemic genotypes do not often lead to treatment failure in the absence of other risk factors. Cirrhosis and previous treatment failure are the most common risk factors associated with treatment resistance; however, it is not yet fully established if such risk factors are invariably present in DAA failure with endemic lineages. Further clinical trials are required to investigate this.

### *In vitro* susceptibility to DAAs

The *in vitro* efficacy of second-generation NS5A inhibitors, pibrentasvir and velpatasvir, for infectious clones of genotypes 1a, 1b, 2a, 3a, 4a, 5a, 6a and 7a is high, in keeping with the pan-genotypic activity reported *in vivo* (with the exception of 7a for which a single case report of SVR following sofosbuvir and velpatasvir is available).^2^^,^^3^^,^^37^ The presence of RASs is only occasionally associated with treatment failure in such subtypes, often in patients with cirrhosis.^2^

A recent *in vitro* study investigated the efficacy of first and later generation NS5A inhibitors daclatasvir, elbasvir, ledipasvir, pibrentasvir and velpatasvir against several endemic subtypes (1l, 3b, 3g, 4r, 6u and 6v) using the JFH1 sub-genomic replicon (SGR-JFH1) backbone and the NS5A gene of the respective genotype of interest. In this evaluation, only pibrentasvir had high-level pan-genotypic activity *in vitro*.^34^ NS5A sequences of genotypes 1l and 4r typically encode a 28M/30R/31M motif at positions that are associated with a resistant phenotype *in vivo*.^5^ The NS5A Y93H mutation is also commonly found in genotype 4r-infected patients in whom treatment has failed.^5^^,^^6^ Subtypes 3b and 3g invariably encode the double NS5A RAS 30K/31M. This double combination has been shown to confer high-level drug resistance when introduced into the 3a subtype, suggesting that subtypes 3b and 3g are more likely to reduce the effectiveness of NS5A inhibitors.^38^ The NS5A sequences of subtypes 6u and 6v encode the 28V/30S/93S triple polymorphism in 100% (2/2 and 6/6) of published 6u and 6v sequences. This triple motif is associated with substantially reduced effectiveness of ombitasvir for genotype 6a.^39^ In the SGR-JFH1 backbone model (containing genotype-specific NS5A), subtypes 3b and 3g were resistant to daclatasvir, elbasvir, ledipasvir and velpatasvir, while 1l, 4r, 6u and 6v were sensitive to elbasvir and velpatasvir but not to daclatasvir and ledipasvir.^34^ Clinical trials are indicated to evaluate the response to treatment of these subtypes in areas where they occur commonly.

**Key point**

Clinical trials have involved treatment of HCV-infected individuals in high income countries where epidemic lineages of HCV are the most prevalent circulating virus. Some endemic lineages, for example 1l and 4r, are known to respond less well to direct-acting antiviral treatment than epidemic lineages.

### Clinical trial and real-world experience with DAAs

Clinical studies have predominantly been conducted in HICs where endemic subtypes occur occasionally, usually in cases of imported infection. Many large clinical trials in HICs have failed to identify subtypes within treatment arms making interpretation challenging. Clinical trials in LMICs have been carried out in a small number of countries. These have been observational and have usually involved only small numbers of patients without the benefit of sequence analysis for the detection of resistance.

Some genotype 1 subtypes prevalent in West Africa have been associated with lower-than-expected SVR rates to current DAA regimens. Two studies in the UK have shown that genotype 1l (prevalent in Nigeria and Cameroon) and several unclassified genotype 1 subtypes are associated with lower-than-expected SVR rates.^4^^,^^5^ In 1 UK study, carried out in an area with a high number of patients originating from West Africa, only 75% of African patients infected with genotype 1 subtypes rarely found in the UK achieved SVR, whereas a high rate of response was achieved in those infected with genotypes 1a and 1b.^4^ Studies in West Africa are awaited.

Evidence confirming the *in vitro* resistance profile of some genotype 3 subtypes has also emerged recently in clinical studies. In a Chinese single-arm phase III trial, 89% of patients without cirrhosis with genotype 3b HCV responded to treatment with sofosbuvir and velpatasvir while only 50% of those with cirrhosis achieved SVR.^17^ Trials of patients infected with genotype 3g are awaited.

The SHARED study was a single-arm prospective evaluation of the efficacy and safety of ledipasvir/sofosbuvir in adults diagnosed with chronic HCV genotype 4 infection in Rwanda.^7^ Treatment with ledipasvir and sofosbuvir led to high SVR rates of 93% in patients with the highly prevalent genotype 4k variant found in Gabon, the DRC, Uganda and Rwanda; a generic formulation of this treatment has been widely distributed in sub-Saharan Africa. Patients with genotypes 4q and 4v also responded well to treatment, with SVR rates of 90% and 100%, respectively). In contrast, as predicted *in silico*, *in vitro* and in small numbers of patients in European trials, genotype 4r was associated with a far lower-than-expected SVR response to ledipasvir/sofosbuvir of only 56% (27/48 individuals). The SVR12 response rate among all patients in the trial with a non-subtype 4r infection was 93% (234/252 of those treated). Genotype 4r is prevalent in several countries in sub-Saharan Africa, including the DRC, Rwanda, South Africa, Ethiopia and Malawi and has been detected in Saudi Arabia. Genotype 4r NS5A resistance-associated polymorphisms (to daclatasvir, velpatasvir and ledipasvir-based regimens) have also been reported in small numbers of participants in studies in the UK and France.^5^^,^^6^^,^^40^ A phase IV follow-on study is ongoing in Rwanda (SHARED3) to investigate the use of sofosbuvir and velpatasvir as first-line and sofosbuvir/velpatasvir and voxilaprevir as second-line therapy. Genotypes 4b (also found in the DRC), 4c and 4g (found in the DRC and Gabon) and 4l were also detected and treated in the SHARED study but were present in very small numbers. Very little or no SVR data are available for other genotype 4 subtypes not detected in this study, including 4e (Gabon), 4f (Cameroon, Algeria), 4h (the DRC), 4m (Egypt), 4n (Egypt), 4o (Saudi Arabia), 4p (Cameroon), 4s (Uganda) and 4t (Cameroon).^41^ Of these, subtype 4b has shown evidence of resistance to ledipasvir *in vitro* and merits further study.^35^

Treatment responses of genotype 6a have also been well documented both *in vitro* and *in vivo* but other genotype 6 subtypes have not.^37^^,^^42^ Limited data from clinical trials have highlighted high SVR12 rates for genotype 6a.^2^^,^^3^ It will be important to identify response rates to other genotype 6 subtypes where they are prevalent, particularly in Southeast Asia. Given the presence of a triple RAS motif in subtypes 6u and 6v and the results of *in vitro* assays with first-generation NS5A inhibitors daclatasvir and ledipasvir, real-world studies and clinical trials using these and other agents (including the generic NS5A inhibitor ravidasvir) are eagerly anticipated.

**Key point**

We advocate for further studies to characterise the prevalence and genetic diversity of endemic HCV lineages in low middle-income countries to determine optimal regional treatment strategies and facilitate the World Health Organization’s 2030 elimination strategy.

### Mis-genotyping by commercial diagnostic assays

As well as affecting treatment outcomes, genetic variation may also result in difficulties with genotyping HCV subtypes. While an in-depth discussion and summary of the evidence behind the occurrence of mis-genotyping HCV by commercial assays is beyond the scope of this review, we highlight the importance of this issue, particularly when considering the implications for treatment. For example, in the SHARED study, several subtype 4r samples were mis-typed by an Abbott assay as genotype 1,^7^ this assay was also associated with mistyping of genotype 6 subtypes as genotype 1^43^ and genotype 7 HCV as genotype 2.^44^

## Implications and future planning

Many genotypes that have not been assessed in large randomised controlled clinical trials also appear to respond well to treatment in smaller real-world studies.^2^^,^^7^^,^^45^^,^^46^ However, recent *in vitro* experimental and clinical data have shown that some sub-genotypes are associated with a reduced susceptibility to current DAA therapies.^2^^,^^34^ Several genotypes common in parts of sub-Saharan Africa and Asia, including genotypes 1l, 3b, 3g, 4b, 4r, 6u and 6v harbour RASs and have evidence of variable levels of resistance to NS5A inhibitors *in vitro*. Genotypes 1l, 3b and 4r have also been found to respond less well than expected to some DAAs in clinical studies. Genotypes 7 and 8 have not yet been assessed in clinical trials. These genotypes are not rare in affected countries but are most prevalent in LMICs where large scale clinical trials have not taken place due to the absence of highly profitable markets. Future international regulation and/or political pressure on the drug industry may be required to ensure that the WHO HCV elimination target is achieved and policymakers, patients and treating physicians should recommend that pharmaceutical companies based in HICs consider the ethics of access to effective treatment for all in addition to market forces when developing new therapies.

Treating physicians in HICs should consider the possibility of atypical genotypes in patients who may have become infected with HCV in sub-Saharan Africa and Asia – this consideration is now outlined in the most recent EASL guidelines.^2^ Those practicing in countries where such genotypes are common in the population should preferentially use WHO-recommended regimens that include NS5A inhibitors with a higher barrier to resistance, such as glecaprevir/pibrentasvir or sofosbuvir/velpatasvir in preference to first-generation inhibitors such as daclatasvir or ledipasvir. Sofosbuvir/daclatasvir remains a recommended first-line “pan-genotypic” regimen in the current WHO treatment guidelines^3^ – this combination may be highly effective in some countries but may not be optimal in others. Retreatment options for endemic subtypes have only been partially assessed, typically in very small numbers of patients. Current guidelines and some small studies suggest that glecaprevir/pibrentasvir/sofosbuvir or sofosbuvir/velpatasvir/voxilaprevir with rare genotypes may be a suitable option for retreatment in individuals who have undergone unsuccessful therapy with NS5A inhibitors.^2^^,^^5^^,^^6^^,^^47^

Development of treatment registries in LMICs would be of considerable value in tracking real-world responses to treatment and in quantifying the risk of onward transmission of resistant strains. Sub-genotyping has not always been carried out or reported in many clinical trials. While genotyping is not necessary at the individual level, population-level genotyping studies would help individual countries to set up appropriate local treatment strategies. Full genome sequencing can be carried out at relatively low-cost using technologies such as Illumina and Oxford nanopore. The highly portable Oxford nanopore MinION platform (the size of a mobile phone) has been used to generate full genome sequences in virus outbreaks all around the world, including in low-income countries such as the DRC.^48^ Such work has not yet been applied to HCV on a large scale and genetic sequence data is missing from many LMICs. However, it is feasible and should be considered.

Of the 71 million people living with HCV in the world, sub-Saharan Africa,^49^ South and Central America and Asia, are home to 10, 3.5 and 31 million people with HCV, respectively, thus sharing a significant proportion of the burden of HCV infection.^50^ Careful consideration needs to be given to treatment strategies for HCV in these regions – elimination is within our grasp, but one size does not necessarily fit all.

### Abbreviations

DAA, direct-acting antiviral; HCV, hepatitis C virus; HICs, high-income countries; LMICs, low middle-income countries; MSM, men-who-have-sex-with-men; PWID, people who inject drugs; RAS(s), resistance-associated substitutions; SVR, sustained virological response; WHO, World Health Organisation.

## Financial support

10.13039/501100000265Medical Research Council (MRC) (MC_UU_12014/1) and 10.13039/100004440Wellcome Trust (102789/Z/13/A).

## Authors’ contributions

E.T. is the corresponding author and devised the main conceptual ideas as well as the outline of the review article. E.T. wrote the manuscript supported by R.S. and L.A. R.S. and L.A. gathered and curated the data and analysed the genomic data. M.N, J.S. and R.S. created the data visualisation figures.

## Conflict of interest

The authors declare that there is no conflict of interest.

Please refer to the accompanying ICMJE disclosure forms for further details.
