# Supplementary material for: Non-epidemic HCV genotypes in low- and middle-income countries and the risk of resistance to current direct-acting antiviral regimens
Source: J Hepatol. 2021 Aug;75(2):462–73. doi: 10.1016/j.jhep.2021.04.045 (PMC8310923; doi:10.1016/j.jhep.2021.04.045)
Supplement: Multimedia component 1 [file mmc1.pdf]

Non-epidemic HCV genotypes in low- and middle-income countries and the risk of resistance to current direct-acting antiviral regimens

Rajiv Shah, Lucrece Ahovegbe, Marc Niebel, James Shepherd, Emma C. Thomson

Table of contents

Table S1 2
Table S2 17
Table S3 24
Supplementary references 32

Supplementary Tables

Accession numbers are provided for the relevant subtypes and whole genome HCV sequences were obtained from the International Committee on Taxonomy of Viruses Flaviviridae study group webpage (https://talk.ictvonline.org/ictv_wikis/flaviviridae/hepacivirus/m/hepacivirus-files).

Substitutions are shaded in different colours that reflect the strength of evidence for drug resistance. Red shaded substitutions have the strongest evidence and are those that have demonstrated an *in vitro* resistance of greater than or equal to level 5 and have been found at baseline or treatment-emergent in vivo OR they have been found at baseline and treatment-emergent *in vivo* and. Orange shaded substitutions are those that have either shown *in vitro* or *in vivo* resistance. Yellow shaded substitutions are those that have shown *in vitro* resistance only.

All analysis of RASs was conducted using the web tool HCV GLUE http://hcv-glue.cvr.gla.ac.uk (1).

Table S1: RASs in the NS5A gene in endemic HCV genotypes

Genotype	Accession Number of Reference genome	Site*										
		24	28	30	31	37	54	58	62	75	92	93
1c	D14853<br>AY051292<br>AY651061	K24K	M/V28M/V	Q30Q	-	L/V37V	H/N54H	-	-	-	-	H/N/Y93H
1d	KJ439768	-	-	R30R	M31M	L37L	-	-	-	-	-	-
1e	KC248194	K/R24K	M28M	Q/R30Q	L/M31M	L37L	H/N54H	P/S58S	-	-	T92T	-
1g	AM910652	-	-	Q/R30R	-	L37L	-	-	-	-	-	F93F
1h	KC248198<br>KC248199	-	-	R30R	-	-	-	-	-	A75A	-	-
1i	KJ439772	-	-	R30R	-	I37I	-	-	-	-	-	-

Genotype	Accession Number of Reference genome	Site*										
		24	28	30	31	37	54	58	62	75	92	93
1j	KJ439773	K24K	M28M	Q30Q	-	L37L	-	-	-	-	-	-
1k	KJ439774	-	A28A	Q30Q	-	I37I	-	-	-	-	-	-
1l	KC248193<br>KC248197<br>KC248196	G/S24G	M28M	Q/R30Q/R	M31M	F/L37L	H/N54H	-	-	A/S75A	-	-
1m	KJ439778<br>KJ439782	K24K	M28M	Q/S30Q	M/V31M/V	F/I37I	-	-	-	-	-	C/H93C/H
1n	KJ439781<br>KJ439775	K24K	-	Q30Q	M31M	I/V37I	-	-	-	-	-	-
1o	KJ439779<br>MH885469	K/Q24K	L/M28M	Q/R30Q	L31M	F/L/V37L	-	-	-	-	-	Y93H

Genotype	Accession Number of Reference genome	Site*										
		24	28	30	31	37	54	58	62	75	92	93
2d	JF735114	S24S	F/L28L	-	-	-	-	-	-	-	-	-
2e	JF735120	S24S	F28F	-	M31M	-	-	-	-	-	S92S	-
2f	KC844042<br>KC844050	F/S24S	F/S28S	-	M31M	-	-	-	-	-	C/S92S	-
2i	DQ155561	S24S	F28F	-	M31M	-	-	-	-	-	-	-
2j	HM777358<br>JF735113<br>HM777359	S24S	F/L28L	-	M31M	-	-	-	-	-	-	-
2k	AB031663<br>JX227953	S24S	F/L28L/I	-	I/M31M	-	-	-	-	-	-	-

Genotype	Accession Number of Reference genome	Site*										
		24	28	30	31	37	54	58	62	75	92	93
2m	JF735111 JX227967	S24S	L28L	-	M31M	-	-	-	-	-	S92S	-
2q	FN666428 FN666429	S24S	L28L	-	L/M31M	-	-	-	-	-	-	-
2r	JF735115	S24S	F28F	-	M31M	-	-	-	-	-	-	-
2t	KC197238	S24S	L28L	-	-	-	-	-	-	-	-	-
2u	JF735112	S24S	F28F	-	M31M	-	-	-	-	-	-	-
3b	D49374 JQ065709	-	-	K30K	M31M	-	-	-	-	-	-	-

Genotype	Accession Number of Reference genome	Site*										
		24	28	30	31	37	54	58	62	75	92	93
3d	KJ470619	-	-	K30K	M31M	-	-	-	-	-	-	-
3e	KJ470618	-	-	K30K	-	-	-	-	-	-	-	-
3g	JX227954<br>JF735123	-	-	K30K	M/V31M/V	-	-	-	-	-	-	-
3h	JF735126<br>JF735121	-	-	-	-	-	-	-	-	-	-	-
3i	FJ407092<br>JX227955	-	-	K/R30K	-	-	-	-	-	-	-	-
3k	D63821<br>JF735122	-	-	K30K	M31M	-	-	-	-	-	-	-

Genotype	Accession Number of Reference genome	Site*										
		24	28	30	31	37	54	58	62	75	92	93
4b	FJ462435	-	-	S30S	-	-	-	P/S58P	-	-	-	H93H
4c	FJ462436	-	-	-	-	-	-	P58P	-	-	-	-
4d	DQ418786	-	-	-	-	-	-	-	-	-	-	-
4f	EF589161<br>EU392175<br>EU392174	-	-	-	-	-	-	P58P	-	-	-	-
4g	FJ462432<br>JX227971<br>JX227963	-	-	-	-	-	-	P58P	-	-	-	H/R/Y93H
4k	EU392173<br>FJ462438<br>EU392171	-	-	-	-	-	-	P58P	-	-	-	-

Genotype	Accession Number of Reference genome	Site*										
		24	28	30	31	37	54	58	62	75	92	93
4l	FJ839870 JX227957	-	-	-	-	-	-	P58P	-	-	-	-
4m	FJ462433 JX227972	-	-	S30S	-	-	-	P/R58P	-	-	-	-
4n	FJ462441 JX227970	-	-	-	-	-	-	P/T58T	-	-	-	-
4o	FJ462440 JX227977	-	M28M	-	-	-	-	P58P	-	-	-	-
4p	FJ462431	-	-	-	-	-	-	P58P	-	-	-	-
4q	FJ462434	-	-	-	-	-	-	P58P	-	-	-	-

Genotype	Accession Number of Reference genome	Site*										
		24	28	30	31	37	54	58	62	75	92	93
4r	FJ462439<br>JX227976	-	M/V28M	R30R	L/M31L	-	-	P58P	-	-	-	-
4s	JF735136	-	-	-	-	-	-	P58P	-	-	-	-
4t	FJ839869	-	-	-	-	-	-	P58P	-	-	-	-
4v	HQ537009<br>JX227959<br>HQ537008<br>JX227960	-	-	-	-	-	-	P58P	-	-	-	-
4w	FJ025855<br>FJ025856	-	M28M	S30S	-	-	-	P58P	-	-	-	S93S
5a	AF064490	-	-	-	-	-	-	-	-	-	-	-

Genotype	Accession Number of Reference genome	Site*										
		24	28	30	31	37	54	58	62	75	92	93
6b	D84262	-	T28T	-	I31I	-	-	S58S	-	-	-	-
6c	EF424629	-	V28V	-	-	-	-	-	-	-	-	-
6d	D84263	-	V28V	-	-	-	-	P/S58S	-	-	-	I/T93I
6e	DQ314805<br>EU246932<br>EU246931	-	M/V28V	S30S	-	-	-	-	-	-	-	-
6f	DQ835760<br>EU246936	-	A/T28A	S30S	-	-	-	P/S58S	-	-	-	-
6g	D63822<br>DQ314806	-	M28M	-	-	-	-	-	-	-	-	-

Genotype	Accession Number of Reference genome	Site*										
		24	28	30	31	37	54	58	62	75	92	93
6h	D84265	-	V28V	-	-	-	-	-	-	-	-	-
6i	DQ835770<br>DQ835762	-	V28V	-	-	-	-	-	-	-	-	-
6j	DQ835769<br>DQ835761	-	V28V	-	-	-	-	-	-	-	-	-
6k	D84264	-	V28V	-	-	-	-	-	-	-	-	-
6l	EF424628<br>JX183556	-	V28V	-	-	-	-	-	-	-	-	-
6m	DQ835767<br>DQ835766	-	V28V	S30S	-	-	-	-	-	-	-	S93S

Genotype	Accession Number of Reference genome	Site*										
		24	28	30	31	37	54	58	62	75	92	93
6n	DQ278894<br>DQ835768<br>EU246938	-	V28V	S30S	-	-	-	-	-	-	-	S93S
6o	EF424627<br>EU246934	-	-	-	-	-	-	A58A	-	-	-	S/T93S
6p	EF424626	-	M/V28V	S30S	-	-	-	-	-	-	-	-
6q	EF424625	-	M/V28V	S30S	-	-	-	-	-	-	-	-
6r	EU408328	-	-	-	-	-	-	-	-	-	-	-
6s	EU408329	-	-	-	-	-	-	-	-	-	-	-

Genotype	Accession Number of Reference genome	Site*										
		24	28	30	31	37	54	58	62	75	92	93
6t	EF632071<br>EU246939	-	V28V	S30S	-	-	-	-	-	-	-	-
6u	EU246940	-	V28V	S30S	-	-	-	-	-	-	-	S93S
6v	EU158186<br>EU798760<br>EU798761	-	V28V	S30S	-	-	-	-	-	-	-	S93S
6w	DQ278892<br>EU643834<br>EU643836	-	F28F	-	-	-	-	-	-	-	-	-
6xa	EU408330<br>EU408332<br>EU408331	-	V28V	S30S	-	-	-	-	-	-	-	-
6xb	JX183552<br>KJ567645	-	V28V	-	-	-	-	-	-	-	-	-

Genotype	Accession Number of Reference genome	Site*										
		24	28	30	31	37	54	58	62	75	92	93
6xc	KJ567651	-	V28V	-	-	-	-	-	-	-	-	-
6xd	KM252789<br>KM252790<br>KM252791	-	F/L28F	-	-	-	-	A/P58A	-	-	-	-
6xe	JX183557<br>KM252792	-	V28V	S30S	-	-	-	-	-	-	-	S93S
6xf	KJ567646<br>KJ567647											
6xg	MH492361<br>MH492360<br>MH492362	-	F/L/V28V	A/R/S30S	-	-	-	-	-	-	-	S/T93S
6xh	MG879000											

Genotype	Accession Number of Reference genome	Site*										
		24	28	30	31	37	54	58	62	75	92	93
7a	EF108306	-	-	-	-	-	-	-	-	-	-	-
7b	KX092342											
8a	MH590698<br>MH590699<br>MH590700<br>MH590701											

For subtypes 6xf, 6xh, 7a and 8a there is not enough known about these genomes to comment on RASs in the NS5A gene.

*Sites occur at the same position in the relevant genotype 1a, 2a, 3a, 4a, 6a, 7a and 8a RefSeq reference sequences.

Table S2: RASs in the NS3 gene in endemic HCV genotypes

Genotype	Accession Number of Reference genome	Site*										
		36	54	56	80	107	122	132	156	166	168	170
1c	D14853<br>AY051292<br>AY651061	-	-	-	-	-	N/T122N	-	-	-	-	V170V
1d	KJ439768	-	S/T54S	-	K80K	V107I	-	I132I	A156A	-	D168D	I/V170V
1e	KC248194	L36L	S54S	-	-	-	N/T122N	-	-	-	-	V170V
1g	AM910652	-	S/T54S	-	-	-	-	-	-	-	-	V170V
1h	KC248198<br>KC248199	-	-	-	-	-	-	-	-	-	-	V170V
1i	KJ439772	-	-	-	-	-	-	-	-	-	-	V170V

Genotype	Accession Number of Reference genome	Site*										
		36	54	56	80	107	122	132	156	166	168	170
1j	KJ439773	-	-	-	-	-	N122N	-	-	-	-	V170V
1k	KJ439774	-	-	-	-	-	T122T	-	-	-	-	I170I
1l	KC248193<br>KC248197<br>KC248196	-	-	-	L80L	-	-	-	-	-	-	I/V170V
1m	KJ439778<br>KJ439782	-	-	-	-	-	S/T122T	-	-	-	-	I170I
1n	KJ439781<br>KJ439775	-	-	F/Y56F	L/Q80L	-	-	-	-	-	-	I170I
1o	KJ439779<br>MH885469	V36L	-	-	-	-	S122T	-	-	-	-	I/V170V
2d	JF735114	-	-	-	-	-	-	-	-	-	-	-

Genotype	Accession Number of Reference genome	Site*										
		36	54	56	80	107	122	132	156	166	168	170
2e	JF735120	-	-	-	-	-	-	-	-	-	-	-
2f	KC844042<br>KC844050	-	-	-	-	-	-	-	-	-	-	-
2i	DQ155561	-	-	-	-	-	-	-	-	-	-	-
2j	HM777358<br>JF735113<br>HM777359	-	-	-	-	-	-	-	-	-	-	-
2k	AB031663<br>JX227953	-	-	-	-	-	-	-	-	-	-	-
2m	JF735111<br>JX227967	-	-	-	-	-	-	-	-	-	-	-
2q	FN666428<br>FN666429	-	-	-	-	-	-	-	-	-	-	-

Genotype	Accession Number of Reference genome	Site*										
		36	54	56	80	107	122	132	156	166	168	170
2r	JF735115	-	-	-	-	-	-	-	-	-	-	-
2t	KC197238	-	-	-	-	-	-	-	-	-	-	-
2u	JF735112	-	-	-	-	-	-	-	-	-	-	-
3b	D49374<br>JQ065709	-	-	Y56Y	-	-	-	-	-	-	Q168Q	I170I
3d	KJ470619	-	-	Y56Y	-	-	-	-	-	S166S	Q168Q	I170I
3e	KJ470618	-	-	Y56Y	-	-	-	-	-	-	Q168Q	I170I
3g	JX227954<br>JF735123	-	-	Y56Y	-	-	-	-	-	S166S	Q168Q	I170I

Genotype	Accession Number of Reference genome	Site*										
		36	54	56	80	107	122	132	156	166	168	170
3h	JF735126<br>JF735121	-	-	Y56Y	-	-	-	-	-	S166S	Q168Q	I170I
3i	FJ407092<br>JX227955	-	-	-	-	-	-	-	-	-	-	-
3k	D63821<br>JF735122	-	-	Y56Y	-	-	-	I/L132I	-	-	Q168Q	I170I
4b	FJ462435	-	-	-	-	-	-	-	-	-	-	-
4c	FJ462436	-	-	-	-	-	-	-	-	-	-	-
4f	EF589161<br>EU392175<br>EU392174	-	-	-	-	-	-	-	-	-	-	-
4g	FJ462432<br>JX227971<br>JX227963	-	-	-	-	-	-	-	-	-	-	-

Genotype	Accession Number of Reference genome	Site*										
		36	54	56	80	107	122	132	156	166	168	170
4k	EU392173<br>FJ462438<br>EU392171	-	-	-	-	-	-	-	-	-	-	-
4l	FJ839870<br>JX227957	-	-	-	-	-	-	-	-	-	-	-
4m	FJ462433<br>JX227972	-	-	-	-	-	-	-	-	-	-	I/V170I
4n	FJ462441<br>JX227970	-	-	-	-	-	-	-	-	-	-	-
4o	FJ462440<br>JX227977	-	-	-	-	-	-	-	-	-	-	-
4p	FJ462431	-	-	-	-	-	-	-	-	-	-	-
4q	FJ462434	-	-	-	-	-	-	-	-	-	-	-

Genotype	Accession Number of Reference genome	Site*										
		36	54	56	80	107	122	132	156	166	168	170
4r	FJ462439<br>JX227976	-	-	-	-	-	-	-	-	-	-	-
4s	JF735136	-	-	-	-	-	-	-	-	-	D/T168T	-
4t	FJ839869	-	-	-	-	-	-	-	-	-	-	-
4v	HQ537009<br>JX227959<br>HQ537008<br>JX227960	-	-	-	-	-	-	-	-	-	-	-
4w	FJ025855<br>FJ025856	-	-	-	-	-	-	-	-	-	-	-

RASs in the NS3 gene are less common and have been shown only for non-epidemic subtypes in genotypes 1, 2, 3 and 4.

*Sites occur at the same position in the relevant genotype 1a, 2a, 3a, 4a, 6a, 7a and 8a RefSeq reference sequences.

Table S3: RASs in the NS5B gene in endemic HCV genotypes

Genotype	Accession Number of Reference genome	Site*					
		206	282	321	445	448	585
1c	D14853<br>AY051292<br>AY651061	-	-	-	-	-	-
1d	KJ439768	-	-	-	-	-	-
1e	KC248194	-	-	-	-	-	-
1g	AM910652	-	-	-	-	-	V585V
1h	KC248198<br>KC248199	-	-	-	F445F	-	-
1i	KJ439772	-	-	-	-	-	-

Genotype	Accession Number of Reference genome	Site*					
1j	KJ439773	-	-	-	-	-	V585V
1k	KJ439774	-	-	I321I	-	H448H	V585V
1l	KC248193<br>KC248197<br>KC248196	-	-	-	-	-	-
1m	KJ439778<br>KJ439782	-	-	-	-	-	-
1n	KJ439781<br>KJ439775	-	-	I321I	-	-	I/V585V
1o	KJ439779<br>MH885469	-	-	-	-	-	-
2d	JF735114	-	S282T	-	-	-	-

Genotype	Accession Number of Reference genome	Site*					
2e	JF735120	-	-	-	-	-	-
2f	KC844042<br>KC844050	-	-	-	-	-	-
2i	DQ155561	-	-	-	-	-	-
2j	HM777358<br>JF735113<br>HM777359	-	S282T	-	-	-	-
2k	AB031663<br>JX227953	-	-	-	-	-	-
2m	JF735111<br>JX227967	-	-	-	-	-	-
2q	FN666428<br>FN666429	-	-	-	-	-	-

Genotype	Accession Number of Reference genome	Site*					
2r	JF735115	-	-	-	-	-	-
2t	KC197238	-	-	-	-	-	-
2u	JF735112	-	-	-	-	-	-
3b	D49374<br>JQ065709	-	-	-	-	-	-
3d	KJ470619	E206E	-	-	-	-	-
3e	KJ470618	-	-	-	-	-	-
3g	JX227954<br>JF735123	-	-	-	-	-	-

Genotype	Accession Number of Reference genome	Site*					
3h	JF735126<br>JF735121	-	-	-	-	-	-
3i	FJ407092<br>JX227955	E/K206E	-	-	-	-	-
3k	D63821<br>JF735122	-	-	-	-	-	-
4b	FJ462435	-	-	-	-	-	-
4c	FJ462436	-	-	-	-	-	-
4f	EF589161<br>EU392175<br>EU392174	-	-	-	-	-	-
4g	FJ462432<br>JX227971<br>JX227963	-	-	I/V321I	-	-	-

Genotype	Accession Number of Reference genome	Site*					
4k	EU392173<br>FJ462438<br>EU392171	-	-	-	-	-	-
4l	FJ839870<br>JX227957	-	-	-	-	-	-
4m	FJ462433<br>JX227972	-	-	-	-	-	-
4n	FJ462441<br>JX227970	-	-	-	-	-	-
4o	FJ462440<br>JX227977	-	-	-	-	-	-
4p	FJ462431	-	-	-	-	-	-
4q	FJ462434	-	-	-	-	-	-

Genotype	Accession Number of Reference genome	Site*					
4r	FJ462439<br>JX227976<br>MH743147<br>MH743149<br>MH743151	-	S282T	I/V321I	-	-	-
4s	JF735136	-	-	-	-	-	-
4t	FJ839869	-	-	-	-	-	-
4v	HQ537009<br>JX227959<br>HQ537008<br>JX227960	-	-	-	-	-	-
4w	FJ025855<br>FJ025856	-	-	-	-	-	-
6l	N/A		S282T				

RASs in the NS5B gene are less common and have been shown only for non-epidemic subtypes in genotypes 1, 2, 3 and 4. For genotype 4r the S282T NS5B RAS is shown among three sequences (MH743147, MH743149, MH743151) that are not provided in the ICTV genome dataset. This is an important RAS in a subtype that is endemic to Central Africa (2, 3). The S282T RAS has also been seen as a treatment emergent mutation in subtypes 2d, 2j and 6l, however the relevant HCV genome sequences have not been published.

*Sites occur at the same position in the relevant genotype 1a, 2a, 3a, 4a, 6a, 7a and 8a RefSeq reference sequences.

Supplementary references

1. Singer JB, Thomson EC, McLauchlan J, Hughes J, Gifford RJ. GLUE: a flexible software system for virus sequence data. BMC bioinformatics. 2018;19(1):532.

2. Fourati S, Rodriguez C, Hézode C, Soulier A, Ruiz I, Poiteau L, et al. Frequent Antiviral Treatment Failures in Patients Infected With Hepatitis C Virus Genotype 4, Subtype 4r. Hepatology (Baltimore, Md). 2019;69(2):513-23.

3. Gupta N, Mbituyumuremyi A, Kabahizi J, Ntaganda F, Muvunyi CM, Shumbusho F, et al. Treatment of chronic hepatitis C virus infection in Rwanda with ledipasvir-sofosbuvir (SHARED): a single-arm trial. The lancet Gastroenterology & hepatology. 2019;4(2):119-26.
